# Evaluation of Azathioprine-Induced Cytotoxicity in an *In Vitro* Rat Hepatocyte System

**DOI:** 10.1155/2014/379748

**Published:** 2014-07-01

**Authors:** Abdullah Al Maruf, Luke Wan, Peter J. O'Brien

**Affiliations:** ^1^Department of Pharmacology & Toxicology, Faculty of Medicine, University of Toronto, Toronto, ON, Canada M5S 1A8; ^2^Leslie Dan Faculty of Pharmacy, University of Toronto, Toronto, ON, Canada M5S 3M2

## Abstract

Azathioprine (AZA) is widely used in clinical practice for preventing graft rejection in organ transplantations and various autoimmune and dermatological diseases with documented unpredictable hepatotoxicity. The potential molecular cytotoxic mechanisms of AZA towards isolated rat hepatocytes were investigated in this study using “Accelerated Cytotoxicity Mechanism Screening” techniques. The concentration of AZA required to cause 50% cytotoxicity in 2 hrs at 37°C was found to be 400 *μ*M. A significant increase in AZA-induced cytotoxicity and reactive oxygen species (ROS) formation was observed when glutathione- (GSH-) depleted hepatocytes were used. The addition of *N*-acetylcysteine decreased cytotoxicity and ROS formation. Xanthine oxidase inhibition by allopurinol decreased AZA-induced cytotoxicity, ROS, and hydrogen peroxide (H_2_O_2_) formation and increased % mitochondrial membrane potential (MMP). Addition of *N*-acetylcysteine and allopurinol together caused nearly complete cytoprotection against AZA-induced hepatocyte death. TEMPOL (4-hydroxy-2,2,6,6-tetramethylpiperidin-1-oxyl), a known ROS scavenger and a superoxide dismutase mimic, and antioxidants, like DPPD (*N*,*N*′-diphenyl-*p*-phenylenediamine), Trolox (a water soluble vitamin E analogue), and mesna (2-mercaptoethanesulfonate), also decreased hepatocyte death and ROS formation. Results from this study suggest that AZA-induced cytotoxicity in isolated rat hepatocytes may be partly due to ROS formation and GSH depletion that resulted in oxidative stress and mitochondrial injury.

## 1. Introduction

Azathioprine (AZA), prodrug of 6-mercaptopurine, is widely used as an immunosuppressant for several diseases such as inflammatory bowel disease (IBD) and autoimmune diseases and following transplantation to avoid organ rejection [1–4]. In most cases, hepatotoxicity is an unpredictable side effect of AZA, whose molecular and pathogenic mechanisms remain unknown [5]. It has even been reported that 3.5% of 173 adult IBD patients developed hepatitis as a consequence of AZA treatment [6]. A variety of histopathologic findings have been observed in AZA-induced hepatotoxicity. Nodular regenerative hyperplasia, venoocclusive disease, peliosis hepatis, sinusoidal dilatation, and perisinusoidal fibrosis have been reported [7–13]. Cholestasis, with or without associated hepatocyte necrosis, has also been reported for these thiopurine drugs in clinical studies [7, 14]. The molecular mechanisms of AZA-induced cytotoxicity using the “Accelerated Cytotoxicity Mechanism Screening” (ACMS) techniques were investigated in this study.

The ACMS methods determine the molecular cytotoxic mechanisms of drugs/xenobiotics when incubated at 37°C for 2 to 3 hours using freshly isolated hepatocytes from Sprague-Dawley male rats. ACMS is a useful tool for identifying the bioactivation or detoxifying pathways of a drug/xenobiotic by comparing the effects of specific enzyme modulators on cell viability induced by the drug/xenobiotic being investigated. A major assumption with ACMS is that high dose/short time (*in vitro*) exposure simulates low dose/long time (*in vivo*) exposure [15]. With 24 halobenzenes, it was found that the relative lethal concentrations required to cause 50% cytotoxicity in 2 hrs at 37°C (LC_50_, according to ACMS) that was determined using hepatocytes isolated from phenobarbital-induced Sprague-Dawley rats* in vitro* correlated with hepatotoxicity* in vivo* at 24 to 54 hrs [16]. Moreover, using these techniques, the molecular hepatocytotoxic mechanisms found* in vitro* for seven classes of xenobiotics/drugs were found to be similar to the rat hepatotoxic mechanisms reported* in vivo* [17]. Our laboratory successfully used ACMS techniques to investigate molecular mechanisms of drugs/xenobiotics-induced cytotoxicity in isolated rat hepatocytes. Recent examples include chlorpromazine [18]; isoniazid [19]; amodiaquine [20]; and polychlorinated biphenyls [21].

AZA has been reported to conjugate with reduced glutathione (GSH) to form 6-mercaptopurine (6-MP), catalyzed by glutathione S-transferases [22–25]. Previous studies performed with rat hepatocyte primary cultures showed that toxic concentrations of AZA (25–250 *μ*M) led to profound intracellular GSH depletion, mitochondrial injury, metabolic activity reduction, decreased adenosine 5′-triphosphate (ATP) levels, and cell death due to necrosis, not apoptosis. Toxic effects were acute and dose-dependent. Hepatocyte death was prevented by GSH or* N*-acetylcysteine, Trolox (6-hydroxy-2,5,7,8-tetramethylchroman-2-carboxylic acid, a vitamin E analogue), high dose of allopurinol (acts as an antioxidant), cyclosporine A, and glycine [24, 25]. Similar effects were observed by Menor and colleagues [5] where AZA (150 *μ*M) decreased the viability of rat hepatocytes and induced intracellular GSH depletion, metabolic activity reduction, and lactate dehydrogenase release. However, the cell death was not accompanied by DNA laddering, procaspase-3 cleavage, or cytochrome c release. AZA caused mitochondrial dysfunction and activation of stress-activated protein kinase pathways leading to necrotic cell death in intact isolated rat mitochondria [5].

Clinically relevant concentrations of AZA (0.5–5 *μ*M) were also found to be toxic to rat hepatocyte cultures and involved oxidative stress, mitochondrial injury, and ATP depletion that led to cell death by necrosis. Allopurinol (xanthine oxidase inhibitor) and Trolox together provided near complete hepatocyte protection from AZA [10]. Xanthine oxidase is proposed to be involved in several steps of AZA metabolism such as in the direct metabolism of AZA to form an inactive metabolite, 1-methyl-4-nitrothioimidazole, in the conversion of AZA to 6-MP, and in the formation of 6-thiouric acid from 6-MP [10, 24, 25]. The possibility that xanthine oxidase may play a role in AZA-induced tissue injury has been raised by the observation that patients taking allopurinol, a xanthine oxidase inhibitor, experienced less nephrotoxicity during rejection episodes after renal transplantation [26].

Thiopurine S-methyltransferase (TMPT) converts 6-MP to 6-methyl mercaptopurine (6-MMP) and elevated 6-MMP levels were reported to be associated with hepatotoxicity (reviewed in [27, 28]). However, several studies reported AZA-induced hepatotoxicity had no relationship with 6-MMP levels [29–31]. AZA-induced myelosuppression and skin reactions were related to thiopurine S-methyltransferase (TPMT) polymorphisms [2, 32]. However, TPMT polymorphisms did not appear to be involved in AZA-induced hepatotoxicity [33].

In this study, we investigated different toxicity routes of AZA towards isolated rat hepatocytes using ACMS techniques. We hypothesize that AZA causes cytotoxicity towards isolated rat hepatocytes by depleting hepatocyte GSH and producing reactive oxygen species (ROS). We also hypothesize that xanthine oxidase is also involved in AZA-induced oxidative stress in rat hepatocytes.

## 2. Materials and Methods 

### 2.1. Chemicals

Type II collagenase was purchased from Worthington Biochemical Corp. (Lakewood, New Jersey, USA). 4-(2-Hydroxyethyl) piperazine-1-ethanesulfonic acid (HEPES) was purchased from Boehringer-Mannheim Ltd. (Montreal, Canada). Azathioprine and all other chemicals were purchased from Sigma-Aldrich Corp. (Oakville, Ontario, Canada).

### 2.2. Animal Treatment and Hepatocyte Preparation

Male Sprague-Dawley rats weighing 275–300 g (Charles River Laboratories International Inc., USA) were used for experimental purposes and carried out in compliance with the* Guide to the Care and Use of Experimental Animals* [34]. Rats were housed in ventilated plastic cages. There were 12 air changes per hr, 12 hr light photoperiod (lights on at 08:00 hr), and an environmental temperature of 21–23°C with a 50–60% relative humidity. The animals were fed a normal standard chow diet and water* ad libitum*. Hepatocytes were isolated from rats by collagenase perfusion of the liver [35]. Isolated hepatocytes (10 mL, 10^6^ cells/mL) were suspended in Krebs-Henseleit buffer (pH 7.4) containing 12.5 mM HEPES in continually rotating 50 mL round bottom flasks, under an atmospheric condition of 95% O_2_ and 5% CO_2_ in a 37°C water bath for 30 min prior to the addition of chemicals.

GSH-depleted hepatocytes were obtained by preincubating the hepatocytes with 200 *μ*M 1-bromoheptane for 30 min [36]. 1-Bromoheptane rapidly conjugates hepatocyte GSH without affecting hepatocyte viability. GSH precursors,* N*-acetylcysteine (1 mM) or* L*-cysteine (1 mM), were added 30 min prior to the addition of AZA or other agents. Xanthine oxidase-inhibited hepatocytes were obtained by preincubating hepatocytes with 20 *μ*M allopurinol for 30 min [37]. The concentrations of enzyme modulators/antioxidants/ROS scavenger used in the experiments did not affect hepatocyte viability.

### 2.3. Cell Viability

Hepatocyte viability was assessed microscopically by plasma membrane disruption as determined by the trypan blue (0.1% w/v) exclusion test [35]. Hepatocyte viability was determined every 30 min during a 3 hr incubation period. Only cell preparations with viability of 80 to 90% were used.

### 2.4. ROS Formation Assay

Hepatocyte hydroxyl, peroxyl, and other ROS generations were determined using 2′, 7′-dichlorofluorescein diacetate (DCFD) which can permeate hepatocytes and be deacetylated by intracellular esterases to form nonfluorescent dichlorofluorescein. Dichlorofluorescein is oxidized by intracellular ROS to form the highly fluorescent dichlorofluorescein. ROS formation was assayed by withdrawing 1 mL hepatocyte samples at 30 min, which were then centrifuged for 1 min at 5000 ×g. The cells were resuspended in Krebs-Heinseleit buffer and 1.6 *μ*M DCFD was added. The cells were incubated at 37°C for 10 min and the fluorescent intensity (FI) of dichlorofluorescein was measured using a SPECTRAmax Gemini XS spectrofluorometer (Molecular Devices, LLC, CA, USA) set at 490 nm excitation and 520 nm emission wavelengths [38].

### 2.5. Mitochondrial Membrane Potential (MMP) Assay

The uptake of the cationic fluorescent dye, rhodamine 123, has been used for the estimation of MMP according to Andersson and colleagues [39]. Aliquots (500 *μ*L) of the cell suspension at 30 minutes were separated from the incubation medium by centrifugation at 5000 ×g for 1 min. The cell pellet was resuspended in 2 mL of fresh incubation medium containing 1.5 *μ*M rhodamine 123 and incubated at 37°C in a thermostatic bath for 10 min with gentle shaking. Hepatocytes were then separated by centrifugation and the amount of rhodamine 123 remaining in the incubation medium was measured at 490 nm excitation and 520 nm emission wavelengths using a SPECTRAmax Gemini XS spectrofluorometer (Molecular Devices, LLC, CA, USA). The capacity of mitochondria to take up the rhodamine 123 was calculated as the difference in fluorescence intensity between control and treated cells [39] and was expressed as % MMP [40].

### 2.6. Hydrogen Peroxide (H_2_O_2_) Measurement

H_2_O_2_ was measured in hepatocytes by taking samples at 30 min by adding FOX 1 reagent. The FOX 1 reagent consisted of 25 mM sulfuric acid, 250 *μ*M ferrous ammonium sulfate, 100 *μ*M xylenol orange, and 100 mM sorbitol. At the above time point, 50 *μ*L of hepatocytes suspension was added to 950 *μ*L of the FOX 1 reagent and incubated for 30 min at room temperature. Samples were then spectrophotometrically analyzed at 560 nm using a SPECTRAmax Plus 384 spectrophotometer (Molecular Devices, LLC, CA, USA). The extinction coefficient 2.35 × 10^5^ M^−1 ^cm^−1^ was used to measure the concentration of H_2_O_2_ [41].

### 2.7. Cellular GSH and Oxidized Glutathione (GSSG) Determination

GSH and GSSG (the disulfide dimer of GSH) in hepatocytes were determined by commercial kits from Cayman Chemical, MI, USA, according to manufacturer's instruction which utilizes an optimized enzymatic recycling method [42].

### 2.8. Statistical Analysis

The SPSS software package (version 14.0, SPSS Inc., Chicago, USA) was used to analyze the data. Values were expressed as mean ± standard error of the mean (SEM) from 3 independent experiments. Statistical analysis was performed using a one-way analysis of variance (ANOVA) and Tukey's post hoc test to assess significance between control and treatment groups in these experiments. *P* < 0.05 was considered significant.

## 3. Results and Discussion

**Figure 1:**
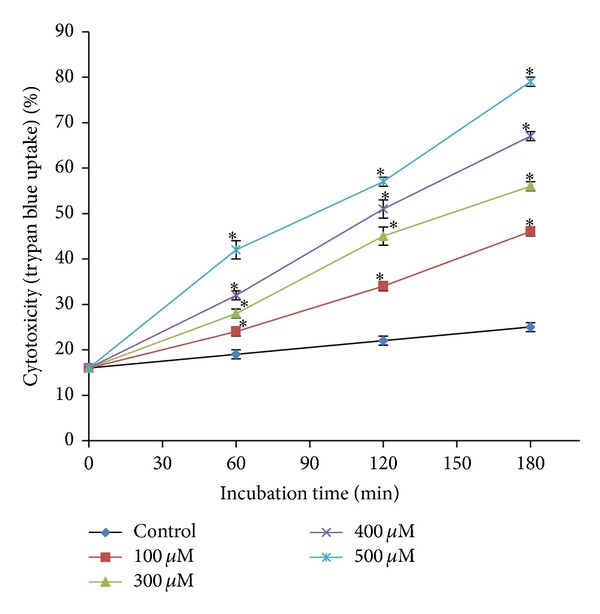
Concentration-response curve of AZA (100–500 *μ*M) towards isolated rat hepatocytes to determine ACMS LC_50_. ∗Significant compared to control hepatocytes.

A concentration and time dependent increase in cytotoxicity and ROS formation and a decrease in % MMP were observed with AZA (100–500 *μ*M) compared to control hepatocytes (Figure 1) incubated for 3 hrs. Incubation of isolated rat hepatocytes for 2 hrs at 37°C with 400 *μ*M AZA induced an approximate 50% loss in hepatocyte viability as measured by the trypan blue exclusion assay (LC_50_, according to ACMS). We use this LC_50_ value to investigate potential cytotoxic mechanisms of drug or xenobiotic under investigation. Although this* in vitro* study is limited for use at high concentrations of the drug, ACMS techniques assume that the drug metabolic/toxic pathways at cytotoxic drug concentrations* in vitro* at 2 hrs are similar to those that occur* in vivo* in 24 to 36 hrs [15].

**Figure 2:**
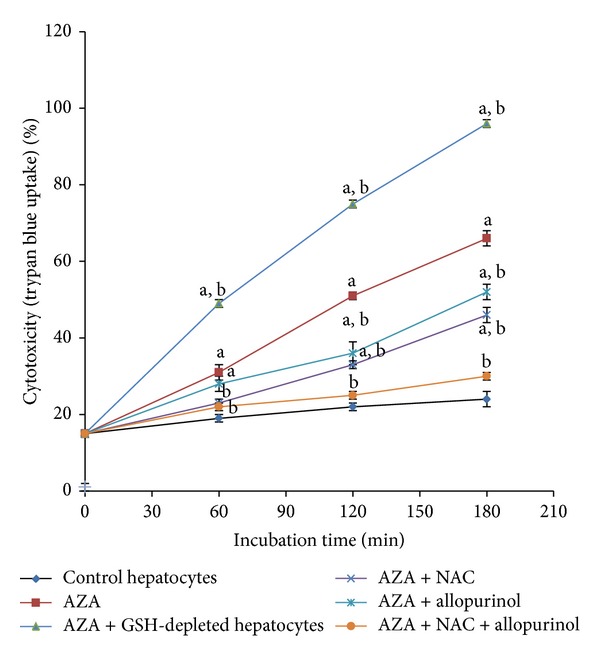
Glutathione and xanthine oxidase dependence of AZA towards rat hepatocytes. NAC,* N*-acetylcysteine; ^a^significant compared to control hepatocytes; ^b^significant compared to AZA (400 *μ*M).

GSH and xanthine oxidase dependence of AZA in isolated rat hepatocytes are presented in Figure 2. AZA treatment (400 *μ*M) significantly depleted hepatocyte GSH and increased GSSG levels (data not shown). A significant increase in AZA-induced cytotoxicity and ROS formation were observed when hepatocyte GSH was depleted by using 1-bromoheptane whereas addition of* N*-acetylcysteine (1 mM, a cysteine precursor which generates GSH) prevented AZA-induced cytotoxicity (Table 1), ROS, and H_2_O_2_ generation and increased % MMP and hepatocyte GSH which indicates that GSH was required for AZA detoxification.* N*-Acetylcysteine has been used as a tool for investigating the role of ROS in numerous biological and pathological processes. The usefulness of* N*-acetylcysteine in different diseases including cardiovascular diseases, cancer, and chemical/metal toxicity has been reviewed in Zafarullah and colleagues [43]. Addition of* L*-cysteine (1 mM) also had similar effects (data not shown) confirming the potential role of GSH in AZA-induced cytotoxicity. A similar depletion of GSH levels and mitochondrial toxicity during AZA metabolism were observed in previous studies in primary cultures of rat hepatocytes with both toxic and clinically relevant AZA concentrations [5, 10, 25]. However, human liver parenchymal cells were reported to be much less sensitive than rat hepatocytes to thiopurine treatments [1]. Protective effects of* N*-acetylcysteine against AZA-induced hepatotoxicity have been reported in several* in vitro* [5, 25, 44] and* in vivo* [45] studies which clearly indicates a potential role of GSH in AZA-induced cytotoxicity.

**Figure 3:**
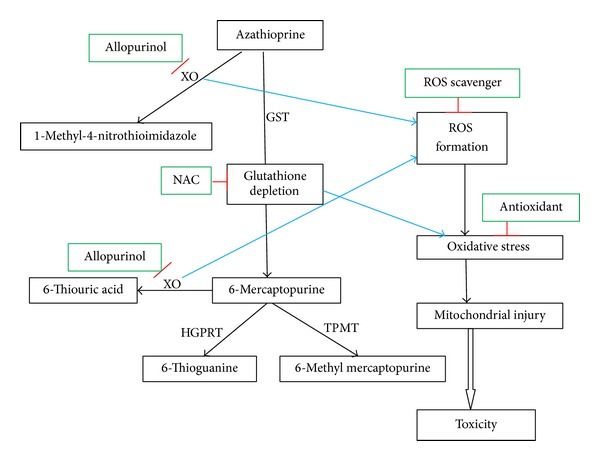
Proposed routes of AZA-induced cytotoxicity in isolated rat hepatocytes. XO, xanthine oxidase; GST, glutathione S-transferase; NAC,* N*-acetylcysteine; HGPRT, hypoxanthine guanine phosphoribosyl transferase; TPMT, thiopurine S-methyl transferase.

Xanthine oxidase is involved in several stages of AZA metabolic pathways (Figure 3) and is well-known to produce ROS [10, 46]. Xanthine oxidase is exemplified by numerous studies in which inhibition of xanthine oxidase attenuated symptoms of several vascular diseases including congestive heart failure, sickle cell anemia, and diabetes [47–49]. When we inhibited xanthine oxidase by preincubating hepatocytes with 20 *μ*M allopurinol, AZA-induced cytotoxicity was significantly prevented (Figure 2). A significant decrease in ROS and H_2_O_2_ formation and an increase in % MMP were observed compared to control hepatocytes with AZA-treated and xanthine oxidase-inhibited hepatocytes (Table 1). Xanthine oxidase inhibition by allopurinol was also found to be protective in primary rat hepatocytes [10]. A recent case study reported that the addition of allopurinol with appropriate AZA dose reduction may correct AZA-induced hepatotoxicity and can induce remission [50]. In AZA or 6-MP nonresponders, addition of allopurinol also demonstrated safety and efficacy for long-term maintenance in IBD patients [51, 52]. In our study, the combined addition of* N*-acetylcysteine (1 mM) and allopurinol (20 *μ*M) showed nearly complete protection against AZA-induced cytotoxicity in rat hepatocytes. The combination of these two agents for improved AZA therapeutic efficacy and liver enzymes needs future clinical experimentation.

A significant decrease in AZA-induced cytotoxicity (Table 2) and ROS formation (Table 1) in isolated rat hepatocytes was achieved by the ROS scavenger and the superoxide dismutase mimic TEMPOL (4-hydroxy-2,2,6,6-tetramethylpiperidin-1-oxyl, 200 *μ*M) suggesting the involvement of ROS. Potent antioxidants, like Trolox (6-hydroxy-2,5,7,8-tetramethylchroman-2-carboxylic acid, 1 mM), DPPD (*N*,*N*′-diphenyl-*p*-phenylenediamine, 2 *μ*M), and mesna (2-mercaptoethanesulfonate, 1 mM), also significantly decreased AZA-induced cytotoxicity (Table 2), ROS, and H_2_O_2_ formation and increased % MMP (Table 1) suggesting the involvement of oxidative stress in AZA-induced cytotoxicity in hepatocytes. Possible routes of cytotoxicity of AZA in isolated rat hepatocytes are presented in Figure 3.

## 4. Conclusions

Data obtained from the ACMS technique suggests that AZA toxicity towards isolated rat hepatocytes involves two distinct pathways (i) a xanthine oxidase-catalyzed production of an inactive metabolite (1-methyl-4-nitrothioimidazole), (ii) glutathione S-transferase- (GST-) catalyzed pathway leading to GSH depletion followed by xanthine oxidase-catalyzed formation of inactive metabolites. Addition of a GSH precursor,* N*-acetylcysteine and a xanthine oxidase inhibitor, and allopurinol together significantly reversed cytotoxicity which raises the possibility of using these two agents therapeutically. Several antioxidants also prevented hepatocyte death suggesting that antioxidant therapy may be of use therapeutically to prevent or decrease AZA-induced hepatotoxicity.* In vivo* animal and clinical studies are warranted to test their therapeutic effectiveness against AZA-induced hepatotoxicity.

## Figures and Tables

**Table 1:** AZA-induced oxidative stress with GSH depletion and protection with a GSH precursor, a xanthine oxidase inhibitor, various antioxidants, and a radical scavenger.

Addition	ROS (FI unit)	MMP (%)	H_2_O_2_ (nmoles/10^6 ^cells)
Incubation time	30 min	30 min	30 min

Control	102 ± 1	100	6.34 ± 0.07
+400 *μ*M AZA	139 ± 3^a^	86 ± 1^a^	8.11 ± 0.08^a^
+GSH-depleted hepatocytes	174 ± 5^a,b^	75 ± 1^a,b^	9.21 ± 0.13^a,b^
+1 mM NAC	124 ± 3^a,b^	89 ± 1^a^	6.97 ± 0.04^a,b^
+20 *μ*M allopurinol	132 ± 3^a^	92 ± 1^a,b^	7.75 ± 0.19^a^
+1 mM NAC + 20 *μ*M allopurinol	107 ± 1^b^	97 ± 2^b^	6.54 ± 0.14^b^
+1 mM mesna	121 ± 3^a,b^	93 ± 2^a,b^	7.10 ± 0.18^a,b^
+1 mM Trolox	113 ± 3^b^	94 ± 2^a,b^	7.12 ± 0.02^a,b^
+200 *μ*M TEMPOL	123 ± 4^a,b^	93 ± 1^a,b^	7.46 ± 0.18^a,b^
+2 *μ*M DPPD	124 ± 2^a,b^	92 ± 2^a,b^	7.26 ± 0.06^a,b^

Data are presented as mean ± SEM (*n* = 3). All modulating agents were noncytotoxic compared to control hepatocytes at concentrations used. Refer to Section 2 for a description of the experiments performed and experimental conditions. FI, fluorescence intensity; NAC, *N*-acetylcysteine; mesna, 2-mercaptoethanesulfonate; Trolox, 6-hydroxy-2,5,7,8-tetramethylchroman-2-carboxylic acid; TEMPOL, 4-hydroxy-2,2,6,6-tetramethylpiperidin-1-oxyl; DPPD, *N,N*′-diphenyl-*p*-phenylenediamine; ^a^significant compared to control (only hepatocytes); ^b^significant compared to 400 *μ*M AZA.

**Table 2:** Effects of a ROS scavenger and various antioxidants on AZA-induced cytotoxicity in isolated rat hepatocytes.

Addition	Cytotoxicity (trypan blue uptake) (%)		
Incubation time	60 min	120 min	180 min

Control hepatocytes	19 ± 1	22 ± 1	24 ± 2
+400 *μ*M AZA	31 ± 2^a^	51 ± 1^a^	66 ± 2^a^
+1 mM mesna	23 ± 1^b^	33 ± 1^a,b^	45 ± 1^a,b^
+1 mM Trolox	24 ± 1^b^	35 ± 2^a,b^	43 ± 1^a,b^
+200 *μ*M TEMPOL	24 ± 1^b^	38 ± 2^a,b^	49 ± 2^a,b^
+2 *μ*M DPPD	26 ± 1^a^	37 ± 1^a,b^	47 ± 1^a,b^

Data are presented as mean ± SEM (*n* = 3); all modulating agents were noncytotoxic compared to control hepatocytes at concentrations used. Refer to Section 2 for a description of the experiments performed and experimental conditions. Mesna, 2-mercaptoethanesulfonate; Trolox, 6-hydroxy-2,5,7,8-tetramethylchroman-2-carboxylic acid; TEMPOL, 4-hydroxy-2,2,6,6-tetramethylpiperidin-1-oxyl; DPPD, *N,N*′-diphenyl-*p*-phenylenediamine. ^a^Significant compared to control (only hepatocytes); ^b^significant compared to 400 *μ*M AZA.
